# Hospital costs and burden of disease due to yellow fever re-emergence in South America: a Colombian analysis

**DOI:** 10.1016/j.ijregi.2026.101040

**Published:** 2026-10-13

**Authors:** Deyna Ripe, Sebastian Alvarez, Mauricio Javier Vera Soto, Nataly Baez, Alan Pirazan, Juan Giraldo

**Affiliations:** 1 Subdirección de enfermedades transmisibles, Ministerio de Salud y Protección Social, Bogotá, Colombia; 2 Department of Medicine, El Bosque University, Bogotá, Colombia; 3 Department of Medicine, Juan N. Corpas University Foundation, Bogotá, Colombia

**Keywords:** Yellow fever, Arboviral disease, Hospital costs, Outbreak, Years of life lost, Premature mortality

## Abstract

**Objectives:**

Yellow fever (YF) is an endemic disease in regions of South America that has recently re-emerged in non-endemic areas. To the best of our knowledge, evidence on the hospital costs and burden of YF remains scarce worldwide. This study is the first analysis of the direct hospital costs and burden of disease due to YF in Colombia.

**Methods:**

A retrospective cross-sectional study was conducted to estimate direct hospital costs and burden of disease due to YF in Colombia during 2025. Direct hospital costs were estimated using a case-based bottom-up costing approach stratified by disease severity according to the Pan American Health Organization Clinical Management and National Guidelines, whereas the burden of disease was assessed using years of life lost (YLLs).

**Results:**

During 2025, 125 confirmed YF cases and 45 deaths were identified in Colombia, with a fatality rate of 36%. The total outbreak-related hospital costs were estimated at 1.0 million US dollars, and 1816.98 YLLs were documented, predominantly affecting rural adults aged 27-60 years.

**Conclusion:**

The re-emergence of YF in Colombia was associated with substantial hospitalization burden due to the high proportion of moderate and severe cases, resulting in increased demand for high-complexity health care services and high-cost advanced therapies. The concentration of YLLs among working-age adults highlights the disease’s social and economic impact.

## Highlights

• A total of 125 confirmed yellow fever cases and 45 deaths were identified in Colombia, with a fatality rate of 36%.
• Yellow fever re-emergence generated over 1 million US dollars in hospital costs.
• More than 1,816 years of life lost were identified, concentrated among individuals aged >60 years.
• Cases and deaths were concentrated in rural working-age populations.
• A total of 66% of cases were classified as clinical severity group C, whereas only 4% belonged to group A.

## Introduction

Yellow fever (YF) continues to be an endemic viral disease. Worldwide, approximately 200,000 cases and 30,000 deaths are estimated annually. Up to 15% of cases develop the severe form of the disease, characterized by hepatic and renal dysfunction, with a fatality rate ranging from 20% to 50%. In this phase, although no established antiviral treatment is available, supportive interventions associated with high hospital costs, such as renal replacement therapy and plasmapheresis, have been required and 30 hepatic transplants have been reported [[1], [2], [3], [4]].

In 2024, 61 cases of YF were confirmed in the Americas; Colombia reported 23 cases, followed by Peru (19), Brazil (eight), Bolivia (eight), and Guyana (three) [5]. In 2025, 346 cases were reported, including 125 in Colombia, 120 in Brazil, and 49 in Peru. Pan American Health Organization (PAHO) and publications from Colombia, Brazil, and Peru support the re-emergence of YF and express concern over a recent expansion to non-endemic areas of South America, suboptimal vaccination coverage, and low hospital response capacity [[6], [7], [8], [9]].

Between 2010 and 2023, the reported YF incidence in Colombia remained low, with approximately one case reported per year. This period coincided with vaccination strategies targeting populations at increased risk, including travelers to endemic Amazonian areas, forestry workers, and farmers. However, the recent increase in cases prompted the issuance of a national epidemiologic alert in 2024, demonstrating changes in transmission dynamics and infections in regions where the disease was previously not present [[10], [11], [12]].

One of the most studied arboviral diseases concerning direct hospital costs is dengue. The cost per hospitalization in middle-income countries ranges between 300 US dollars (USD) for general hospitalization and 9,000 USD in intensive care units (ICUs) for severe dengue in 2023 [13]. In dengue, approximately 35% of patients require hospitalization, whereas in YF, this proportion exceeds 50%, suggesting a potentially greater economic burden than that of other arboviral diseases [11,14,15].

Evidence on the direct hospital costs and burden of disease associated with YF is scarce. A literature search was conducted in PubMed/MEDLINE, Scopus and Google Scholar without language or date restrictions using combinations of the following terms: (“yellow fever” OR “YF”) AND (“hospital cost” OR “direct cost” OR “healthcare cost*” OR “cost of illness” OR “economic burden” OR “burden of disease” OR “years of life lost” OR “YLL”), but no studies that estimated direct hospital costs and years of life lost (YLLs) due to YF were identified. Most economic studies on YF focus on cost-effectiveness analyses of promotion and prevention strategies such as vaccination.

This study aimed to estimate the direct hospital costs and burden of disease due to YF in a re-emergence scenario in Colombia during 2025, with the aim of informing public health decision-making and health care resource planning during outbreaks, particularly in settings where severe cases may require high-complexity and costly hospital care.

## Methods

### General description

A retrospective cross-sectional study was conducted to estimate direct hospital costs and the burden of disease, expressed in YLLs, associated with YF in Colombia during 2025. Cost data were obtained from the Integrated Social Protection Information System (SISPRO), based on the Individual Records of Health Services Provision (RIPS), by identifying cases using International Classification of Diseases, Tenth Revision codes (A95.0, A95.1, and A95.9). Clinical and demographic variables were obtained from the confirmed case database of the National Institute of Health (INS) for 2025. The databases are of “open access” and do not contain identifiable patient information; therefore, this research was classified as without risk.

### Databases and variables

Direct costs from SISPRO database included billed values for diagnosis (first-time consultation and/or follow-up), non-surgical procedures (laboratory tests and diagnostic imaging), and treatment (medicines, supplies, transfusion medicine, and surgical procedures) for the year 2025, the variables collected were institutional final transaction maximum sale price. Non-medical direct costs (transfers and family costs) were excluded (Supplementary Figure S1a and S1b). The INS database (date of consultation, date of hospitalization, date of discharge, or date of death), as well as sociodemographic variables (age, sex, occupation, department, and area of origin), were included, excluding cases outside of Colombia (Supplementary Figure S2). Data on age, sex, and clinical outcome were complete. Severity was classified based on symptoms at admission recorded in the INS database into groups A (mild), B (moderate), C without plasmapheresis (severe), and C with plasmapheresis, according to the PAHO Clinical Management guide and National Guidelines, based on the criteria presented in Table 1 [16,17].

**Table 1:** Clinical classification of YF according to disease severity.

Group A	Group B	Group C
Fever	Mild dehydration	Jaundice
Mild abdominal pain	Vomiting	Melena
Nausea	Diarrhea	Hematemesis
	Persistent abdominal pain	Oliguria
	Mild bleeding (epistaxis, gingival bleeding, petechiae)	Hemodynamic instability (vasopressor use)
	Pregnancy	Seizures
	Hepatic dysfunction without liver failure (ALT/AST >500 U/L, platelets <100,000, INR >1.5)	Respiratory failure (PaO₂ <60 mmHg, mechanical ventilation, FiO₂ ≥35%)
		Mental confusion
		Tachypnea
		Hypotension
		Sings of hypoperfusion
		Arrhythmias
		Acute liver failure (ALT/AST >500 U/L, INR >1.5, encephalopathy)
		Acute renal injury
		Neurological alterations (West Haven ≥2)
		Active bleeding

Abbreviations: ALT, alanine aminotransferase; AST, aspartate aminotransferase; FiO₂, fraction of inspired oxygen; INR, international normalized ratio; PaO₂, partial pressure of oxygen; YF, yellow fever.

Technical parameters and clinical criteria are described in the guidelines for the comprehensive clinical care of yellow fever in Colombia. Version 2. Subdivision of Communicable Diseases, Integrated Management Group for Endemic-Epidemic Diseases. Bogotá: Ministry of Health and Social Protection; 2025.

### Data analysis

Categorical variables are reported as absolute and relative frequencies and continuous variables as median and interquartile range. The SISPRO and INS databases were analyzed separately, information from both sources was combined only at the aggregate level within the costing model. The median days of hospitalization were calculated in Jamovi 2.3.28 software for groups A, B, and C only for laboratory-confirmed cases with available hospitalization and discharge or death dates. Cases with incomplete data were excluded from the length-of-stay calculation. The group-specific median length-of-stay estimates were subsequently incorporated into the costing model to estimate the total cost for each severity group and, subsequently, the overall estimated cost attributable to the outbreak. Among patients who underwent plasmapheresis, the median number of sessions performed per day was calculated using Jamovi version 2.3.28 (The jamovi project, Sydney, Australia; may 25, 2023) and was three sessions per day, consistent with the number of sessions reported in the literature as part of effective intensive plasma exchange regimens [3].

### Direct hospital costs analysis

Direct hospital costs were estimated from the perspective of the Colombian health system using a case-based bottom-up costing approach. For each previously defined clinical severity group (A, B, and C), a representative model patient was constructed according to the health care resources expected for that level of severity, based on the PAHO and Colombian national clinical management guidelines [18]. For each representative model patient, the quantity of each health care resource required per day was identified according to the corresponding clinical severity category. Each resource quantity was multiplied by its respective unit cost obtained from SISPRO/RIPS, and the resulting resource-specific costs were summed to derive the standardized daily hospital cost for each severity group. The sum of these resource-specific costs yielded the standardized daily hospital cost for a representative patient in each severity group.

The model-based hospital stay cost was estimated by multiplying the standardized daily hospital cost by the group-specific median length of stay observed among laboratory-confirmed cases. For group C, separate standardized cost estimates were calculated for patients managed with and without plasmapheresis because of the additional resources associated with this intervention. Costs attributable to fatal cases were estimated using the same methodology, according to their assigned clinical severity group. Finally, the total direct hospital cost attributable to the outbreak was obtained from the integration of the aggregated costs across all severity groups. Costs were converted to USD using the average market representative rate of the Bank of the Republic of Colombia, 2025 (4,052 colombian peso (COP) perUSD). Discount rates were not applied because the analysis was restricted to a 1-year period. Hospital costs were described using frequency tables and median values, and their geographic distribution was represented using a choropleth map of Colombia.

### YLLs analysis

YLLs due to premature mortality were estimated exclusively among deaths occurring in laboratory-confirmed YF cases using the World Health Organization (WHO) Global Health Estimates 2021 standard loss function [19]. For each death, the standard YLLs corresponding to the age at death were assigned according to the WHO Global Health Estimates reference life table. YLLs were calculated as (YLL = ƩNa × La), where Na represents the number of deaths in an age category and La the corresponding standard YLLs per death.

Total YLLs were obtained by summing the age-specific estimates and were subsequently stratified by age group and sex. The same standard loss function was applied to both sexes. The crude YLL rate was calculated as total YLLs divided by the total Colombian population in 2025 (52,695,952) and multiplied by 100,000. Age-specific YLL rates were calculated by dividing the YLLs in each age group by the corresponding Colombian population in the same age group and multiplying by 100,000. Population denominators were obtained from the 2025 population projections of the Colombian National Administrative Department of Statistics. The distribution of YLLs by age group and sex and age-specific YLL rates are presented using bar charts.

## Results

### Characteristics of confirmed cases

In 2025, 2,571 people attended for suspected YF, accounting for 5,359 health care services or procedures recorded in the SISPRO database and 125 laboratory-confirmed cases were identified in the INS database. Of the confirmed cases, 80.8% were men, 19.2% were women, and 46.7% belonged to the 27-60 years age group. A total of 90.0% of the cases came from rural areas. Half of the cases had agriculture and forestry as their occupation. There were 45 deaths, with a fatality rate of 36.0%. The age group with the highest number of deaths was individuals aged >60 years (51.1%). Among those who died, 86.6% were men, 44 cases were from rural areas, and 55.5% were farmers. In addition, 73.3% of deaths occurred among hospitalized patients, and 14 of these patients received care in an ICU (Table 2).

**Table 2:** Social, demographic, and clinical characteristics of YF in Colombia, 2025.

Characteristics	Cases N = 125 (%)	Deaths N = 45 (%)
Age, years		
0-5	1 (0.80)	1 (2.20)
6-12	0	0
13-18	10 (8.00)	1 (2.20)
19-26	12 (9.60)	5 (11.1)
27-60	59 (47.2)	15 (33.3)
>60	43 (34.4)	23 (51.1)
Sex		
Male	101 (80.8)	39 (86.6)
Female	24 (19.2)	6 (13.3)
Geographical zone		
Rural	119 (95.2)	44 (97.7)
Urban	6 (4.80)	1 (2.20)
Occupation		
Farmer	59 (47.2)	25 (55.5)
Other^a^	44 (35.2)	14 (31.1)
Homemaker	14 (11.2)	3 (6.60)
Forestry worker	8 (6.40)	3 (6.60)
In-hospital	98 (78.4)	33 (73.3)
Intensive care unit	43 (34.4)	14 (31.1)
Severity group		
A	5 (4.00)	N/A
B	37 (29.6)	N/A
C	83 (66.4)	45 (100)

Abbreviations: N/A, not applicable; YF, yellow fever.

a Unemployed, day laborer, machinery operator, or miscellaneous occupations.

The department with the highest number of cases was Tolima (109). When classified by clinical severity group, five patients belonged to group A, 37 patients belonged to group B, and 83 patients belonged to group C. A total of 78.4% of cases required hospitalization, with a median of 2 days (1.00-3.00) for group A, 7 days (6.50-10.0) for group B, and 6 days (3.70-9.00) for group C. It was found that 17 cases had a history of YF vaccination, seven of which had less than 10 days between the date of vaccination and the date of symptom onset.

More than half of the cases received care in high-complexity hospitals, and 43 patients required management in an ICU. Plasmapheresis therapy was performed in 17 cases, ranging from one to three sessions per day. In addition, four patients required repeat medical consultations between day 5 and day 20 after hospital discharge due to persistent symptoms; the most frequent symptoms were abdominal pain and jaundice.

### Direct hospital costs and burden of disease in confirmed YF cases

A direct cost per day of hospitalization for YF was obtained for group A of 209.1 USD, for group B of 331.6 USD, for group C without plasmapheresis therapy of 1,507.0 USD, and for group C with plasmapheresis therapy of 2,117.5 USD. The type of service with the highest cost was clinical laboratory tests and diagnostic imaging (2,360.5 USD), followed by supplies and medications (990.1 USD). The cost per plasmapheresis therapy was 331.0 USD, with a total of 16,881.0 USD (17 cases) (Table 3).

**Table 3:** Direct daily hospital cost per patient and hospital stay cost per patient for yellow fever by clinical severity group in Colombia, 2025.

Characteristics	Group A	Group B	Group C	Overall
Daily hospital cost per patient				
Clinical laboratory tests and diagnostic imaging	167.5	212.7	990.1	2,360.5
Supplies and medications	6.80	90.5	446.4	990.1
Plasmapheresis	N/A	N/A	610.6	610.6
Initial and follow-up consultations	34.9	28.3	56.0	175.2
Surgical procedures	N/A	N/A	14.4	28.9
Hospital stay cost per patient				
Clinical laboratory tests and diagnostic imaging	335.0	1,489.2	5,940.8	13,705.9
Supplies and medications	13.6	633.7	2,678.2	6,003.8
Plasmapheresis	N/A	N/A	3,663.5	3,663.5
Initial and follow-up consultations	69.7	198.2	336.0	940.0
Surgical procedures	N/A	N/A	86.7	173.3

Abbreviation: N/A, not applicable.

Cost in US dollars.

The cost per hospital stay for a patient in group A was 418.3 USD (209.1-627.4), group B was 2,321.1 USD (2,155.3-3,315.9), group C without plasmapheresis therapy was 9041.7 USD (5575.7-13,562.6), and group C with plasmapheresis therapy was 12,705.2 USD (7,834.9-19,057.8), according to the days of hospitalization previously described for each group (Table 3).

The total cost for group A was 3,763.8 USD, group B was 102,268.8 USD, group C without plasmapheresis therapy was 281,627.5 USD, and group C with plasmapheresis therapy was 613,936.1 USD. The department with the highest direct hospital costs was Tolima (898,978.0 USD), followed by Putumayo (29,886.0 USD) and Meta (27,125.2 USD) (Figure 1). The cost attributable to fatal cases was 469,157.3 USD. The total cost of the outbreak was estimated at 1,001,596.2 USD.

**Figure 1:**
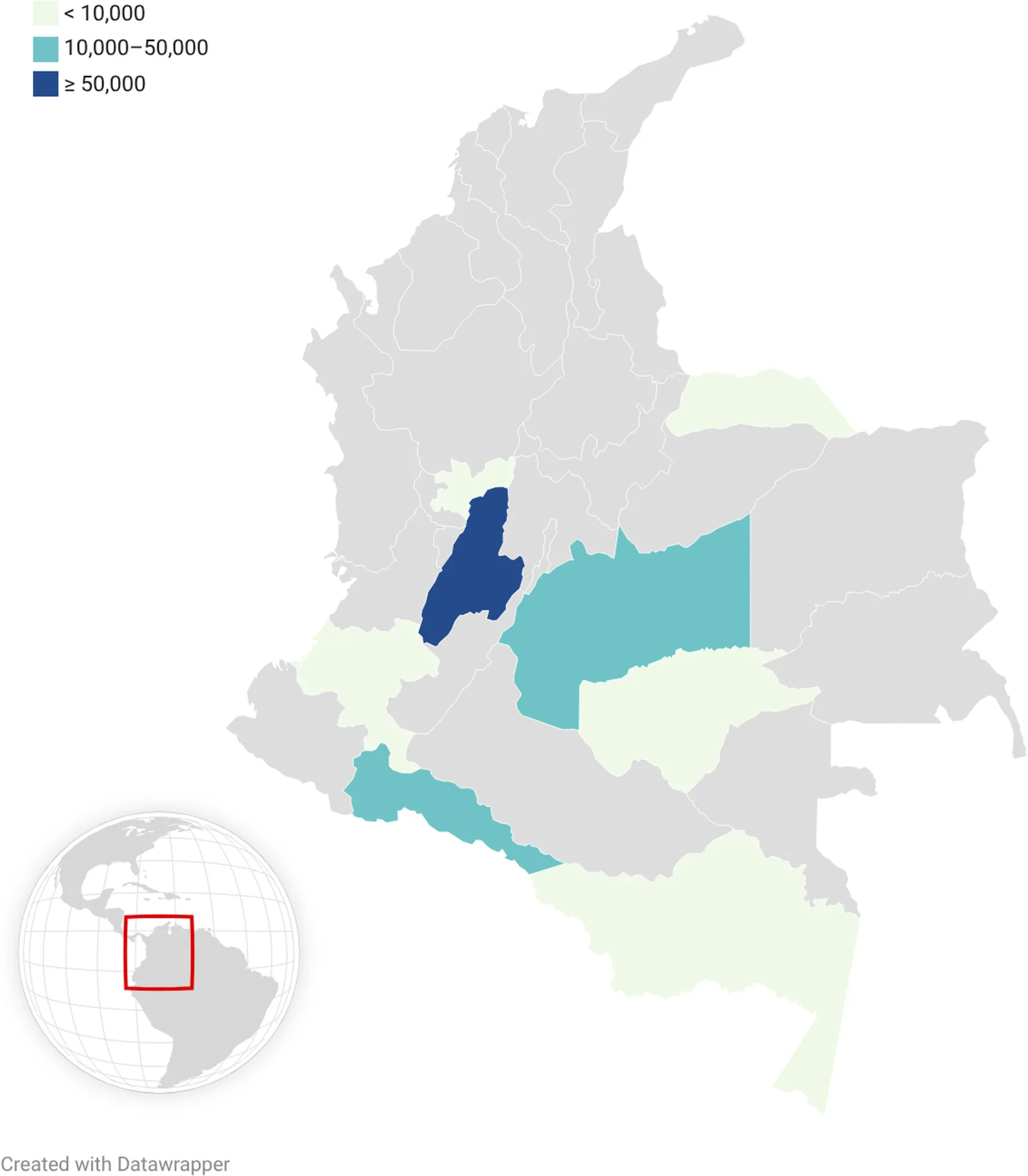
Distribution of yellow fever direct hospital costs by departments, 2025. Costs are presented in US dollars. Gray color: no cases. Data source: Integrated Social Protection Information System and Colombian National Institute of Health.

### Years of life lost due to YF

A total of 1,816.9 YLLs was calculated among laboratory-confirmed YF deaths in Colombia in 2025. Most YLLs occurred among males, who accounted for 1,712.2 YLLs (94.2%), compared with 104.7 YLLs (5.76%) among females. YLLs varied substantially across age groups. The 27-60 years age group accounted for the largest number of YLLs, with 800.8 YLLs (44.1% of the total), followed by individuals aged >60 years, with 500.2 YLLs (27.5%) and those aged 19-26 years, with 351.1 YLLs (19.3%). Lower YLL values were observed among individuals aged 0-5 years (89.7 YLLs; 4.94%) and 13-18 years (75.2 YLLs; 4.14%) (Figure 2a).

**Figure 2:**
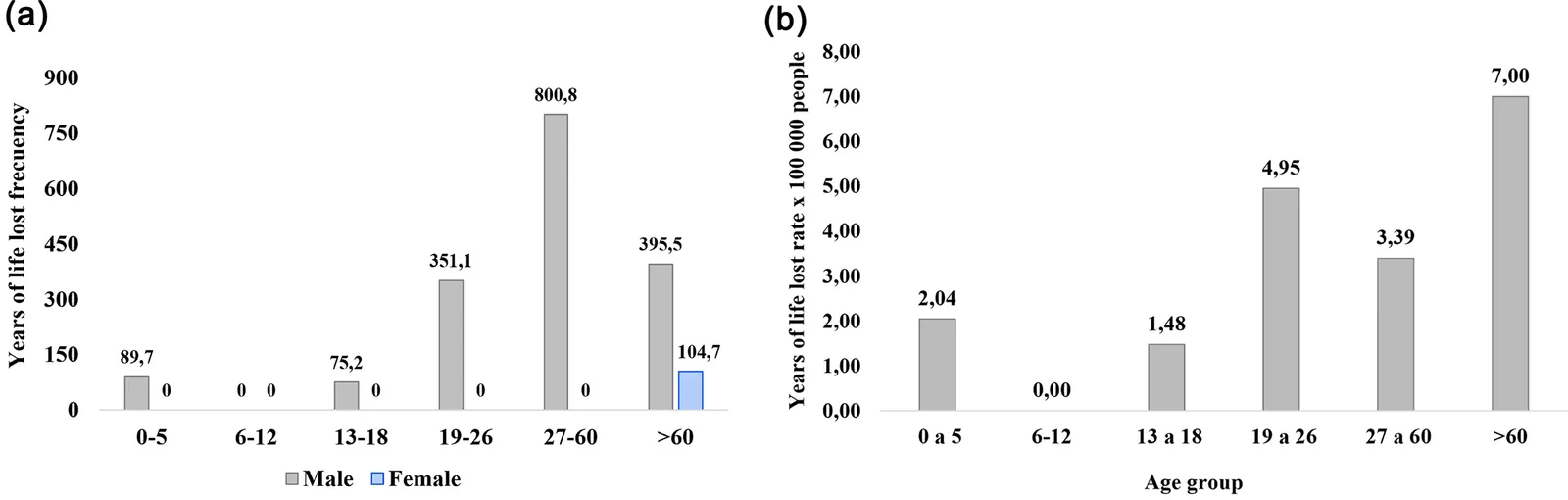
Years of life lost due to yellow fever in Colombia, 2025. (a) Years of life lost by sex and age group due to yellow fever in Colombia. Bars show years of life lost for males (gray) and females (blue). Data are absolute values. Data source: Colombian National Institute of Health database. (b) Years of life lost rate due to yellow fever in Colombia. Rates are expressed per 100,000 population. Data source: Colombian National Institute of Health and Colombian National Administrative Department of Statistics.

The overall YLL rate was 3.45 per 100,000 population. Age-specific YLL rates varied across age groups, with the highest rate observed among individuals aged >60 years (7.00 YLLs per 100,000 population), followed by those aged 19-26 years (4.95), 27-60 years (3.39), 0-5 years (2.04), and 13-18 years (1.48) (Figure 2b).

## Discussion

In this article, we present for the first time the hospital costs and burden of disease due to YF. From the year 2024 to 2025, an increase in YF cases occurred in the Americas. This analysis disaggregates hospital costs by day, hospital stay, and clinical severity. Likewise, the study incorporates the estimation of YLL by sex and age group, broadening the understanding of the economic impact of YF.

Colombia and Brazil presented the highest number of cases in the region. In Colombia, there was an increase in cases of 2300% from 2023 to 2024 and 530% from 2024 to 2025, especially in a region not considered endemic. The expansion of the vector is related to a change in endemic zones due to migratory patterns; climate change; deforestation of the Amazon, Caquetá, and Meta; and the reduction in vaccination during the coronavirus disease 2019 pandemic. This unexpected epidemiologic behavior increases the burden on the health care system, generating an additional, unplanned demand for resources, particularly due to the need for hospital care and management of severe cases. Furthermore, health care personnel in areas that were not previously considered endemic for YF may have limited experience with the disease, which could affect timely case recognition and clinical management [10,11,14].

YF exhibits substantial underreporting in the Americas, with estimates suggesting that only one of every 10 cases is detected, often because case detection is concentrated on severe cases, leading to an underestimation of the true economic burden. In this study, laboratory-confirmed cases represented 4.9% of individuals with YF-coded encounters in SISPRO. As the number of suspected cases increases, the demand for confirmatory testing also increases, underscoring the importance of strengthening clinical suspicion and early diagnostic recognition of YF [20,21].

A relevant finding was the low vaccination coverage even during an outbreak scenario, with levels below the 80% threshold recommended by the WHO for herd immunity [10,22]. In Colombia, the coverage was 64% until July 2024 and 54% in the endemic region (Tolima) until September 2025 [[23], [24], [25]]. These findings underscore the need to strengthen vaccination strategies, particularly in endemic and high-risk areas, and to consider routine vaccination as a key cost-effective measure for preventing future YF outbreaks and reducing their health and economic burden [2].

YF cases and deaths occurred mainly among men, particularly farmers and rural residents, and more than half of the confirmed cases were classified as group C, reflecting the high clinical severity observed and the frequent need for ICU care. The low availability of ICU beds in rural areas and the centralization of high-complexity services necessitate the transfer of patients to capital cities, generating dependence on the referral system and delays in care [[26], [27], [28]]. These characteristics may reflect determinants associated with unfavorable socioeconomic conditions, which limit timely access to health services, favoring progression to severe forms of the disease. The need to optimize timely case confirmation and strengthen the health system’s response, especially in rural areas, is highlighted to reduce delays in access to ICUs [2]. Another novel finding was post-discharge reconsultation in 3% of patients, leading to additional emergency visits and associated health care costs.

Plasmapheresis as a management strategy with recent evidence of a decrease in YF fatality was performed in 17 patients; however, the type of therapy performed and the association with fatality reduction were not considered. The limitations of plasmapheresis are the cost per session, the requirement for specialized infrastructure, and the method used; evidence shows that it should be performed twice a day, with an exchange of the total plasma volume per session associated with an additional infusion of fresh frozen plasma, which complicates its effectiveness [3,29]. Currently, there are no cost-effectiveness studies of plasmapheresis therapy in YF. Furthermore, cost-effectiveness analysis studies of therapeutic interventions are required to guide clinical decision-making and public policy in contexts of limited resources. In addition, no liver transplantation was performed during this outbreak. Previous reports have described a low survival rate of approximately 20% after liver transplantation [4].

Hospital costs for YF increased markedly with clinical severity, ranging from 418.3 USD per hospital stay in group A to 12,705.2 USD in group C patients receiving plasmapheresis. These costs were substantially higher than those previously reported for hospitalized dengue in Colombia and approached or exceeded the upper range of direct medical costs reported for ICU-treated dengue in middle-income countries. However, direct comparisons should be interpreted cautiously because of differences in clinical classification, costing methods, health care settings, and reference years [13,14]. At the outbreak level, direct hospital costs exceeded 1.0 million USD despite having only 125 laboratory-confirmed cases. These findings may suggest that YF re-emergence may impose a considerable financial burden on the health system even when the absolute number of confirmed cases remains relatively limited, although the magnitude of the economic burden may vary according to outbreak intensity, case severity, and health care utilization patterns.

YLLs allow the assessment of the impact of the burden of disease beyond the frequency of fatal events. Although the absolute number and the rate of YLLs observed for YF were lower than those reported for dengue in Colombia, the YLL rate was closer in magnitude to that reported for malaria in other South American settings [30,31]. These differences may partly reflect variations in years, case numbers, transmission patterns, disease incidence, age distribution of deaths, case-fatality rates, and population at risk. In this study, more than 1,500 YLLs were observed. Although the largest absolute number of YLLs occurred among individuals aged 27-60 years, the highest age-specific YLL rate was observed among adults aged >60 years.

The concentration of absolute YLLs among the working-age population may reflect differential exposure to YF in rural occupational settings and activities associated with sylvatic transmission, with potential consequences for productive life and human capital. At the same time, the high YLL rate among adults aged >60 years is particularly relevant in the context of population aging because a growing older population could increase the number of individuals vulnerable to severe disease and fatal outcomes during future outbreaks [32].

Among the study limitations, costs related to hospital infrastructure were excluded because these components could not be reliably traced or standardized. In addition, classification into severity groups A, B, and C was based on available database information and could not be verified through individual clinical chart reviews. Hospital costs were estimated using a standardized case-based costing framework that integrated severity-specific resource-use profiles, unit costs, and observed length of stay without using patient-specific resource consumption data. The estimation of hospital stay costs relied on standardized daily resource use and group-specific median length of stay; therefore, these estimates may not capture patient-level variation in resource utilization or hospitalization duration. Finally, the study relied on administrative databases, which may be subject to incomplete clinical information and coding limitations.

## Conclusion

The re-emergence of YF in Colombia during 2025 resulted in substantial demand for high-complexity health care services, with outbreak-related costs exceeding 1 million USD and a considerable burden of YLLs concentrated among the rural working-age population. More than half of the confirmed cases were classified as group C, highlighting the severity of illness among patients presenting for medical care. These findings underscore the need to strengthen diagnostic capacity and optimize the health system response to future re-emergence scenarios. This study supports public health decision-making by informing prevention and outbreak-response priorities while providing hospitals with evidence for resource planning, budgeting, and preparedness for severe YF cases.

## Funding

This research did not receive any specific grant from funding agencies in the public, commercial, or not-for-profit sectors.

## Ethical approval

The study used anonymized secondary databases without identifiable patient information; therefore, ethics committee approval and informed consent were not required.

## CRediT authorship contribution statement

**Deyna Ripe:** Writing – original draft, Methodology, Investigation. **Sebastian Alvarez:** Writing – review & editing, Visualization. **Mauricio Javier Vera Soto:** Validation, Project administration, Conceptualization. **Nataly Baez:** Supervision, Resources, Investigation. **Alan Pirazan:** Resources, Investigation, Data curation. **Juan Giraldo:** Visualization, Software.

## Declaration of competing interest

The authors have no competing interests to declare.

## Appendix Supplementary materials

Image, application 1
