# Supplementary material for: Hospital costs and burden of disease due to yellow fever re-emergence in South America: a Colombian analysis
Source: IJID Reg. 2026 Oct 13;21:101040. doi: 10.1016/j.ijregi.2026.101040 (PMC13743477; doi:10.1016/j.ijregi.2026.101040)
Supplement: Supplementary file 1 [file mmc1.docx]

**Figure S1.** Flowchart of study population selection and data sources for yellow fever analyses in Colombia, 2025. (a) Information on yellow fever visits was obtained from the Integrated Social Protection Information System database. (b) Information on confirmed cases was obtained from the database of the Colombian National Institute of Health.
